# Commentary: The Role of Neutrophils in the Induction of Specific Th1 and Th17 during Vaccination against Tuberculosis

**DOI:** 10.3389/fcimb.2017.00179

**Published:** 2017-05-12

**Authors:** Patricia Méndez-Samperio

**Affiliations:** Departamento de Inmunología, Escuela Nacional de Ciencias Biológicas, Prol. Carpio y Plan de AyalaMexico City, Mexico

**Keywords:** cellular mechanisms, *M. tuberculosis* infection, neutrophils, Th17 cells, vaccine

Tuberculosis (TB) remains as a global public health problem with an estimated 10.4 million newly emerging active TB cases worldwide in World Health Organization (2016). It is thus a high priority to control *M. tuberculosis (M. tb)* infection. In particular, understanding cellular mechanisms induced by a vaccine against TB will be critical for better TB control. The recent article by Trentini et al. (2016) demonstrated the importance of neutrophils in the development of specific cellular immune responses to a vaccine against TB. According to these authors, this study is the first to demonstrate that the vaccine mc^2^-CMX was shown to protect mice against *M. tb* challenge, mainly due to the specific role of neutrophils in the generation of Th1 and Th17 cells. However, similar research and observations have been published by Trentini et al. (2015) in an earlier study.

The relevance of the important role of Th17 cells in the protection induced by a TB vaccine has recently regained attention. In this regard, the results of Khader et al. demonstrated that in the absence of Th17 cells, the protection induced by a subcutaneous TB vaccine is reduced (Khader et al., 2007). Moreover, Gopal et al. showed that using a mucosal route of vaccination the Th17 cells are important to induce significant protection against *M. tb* in an animal model (Gopal et al., 2013). Furthermore, da Costa et al. demonstrated that the addition of the vaccine mc^2^-CMX to the BCG vaccine induces the generation of Th1 and Th17 cells with higher protective efficacy against TB (da Costa et al., 2014). More recently, the study by Trentini et al. (2016) reported that neutrophil depletion inhibits the Th1 and Th17 cellular generation in the lungs and spleen of vaccinated animals, and that in the absence of these cells the mc^2^-CMX vaccine-induced protection was significantly reduced. Although these authors demonstrated that the presence of IL-17 is important in the induction of Th1- and Th17-cellular responses to increase the protection of the mice against *M. tb*, questions remain whether this research in animal model is really effective in the nature of the human cellular immune response required for protection against infection with *M. tb*.

To date, the critical role of Th cells in protection against TB has been shown in human genetic studies or given that reduced CD4+ T cell numbers in HIV patients lead to dramatically increased susceptibility to TB. Furthermore, vaccine efficacy to protect against TB is evaluated measuring cytokines such as IFN-γ and IL-12. However, combination of these biomarkers cannot provide a correlate of significant protection against human TB and a combination of biomarkers for TB vaccine efficacy remains to be identified (Méndez-Samperio, 2016). In this regard, besides the induction of Th17, natural killer cells must be activated following vaccination because they secrete large quantities of IFN-γ. Simultaneously, potent CD8+ cytolytic T lymphocytes must be activated to kill *M. tb*-infected macrophages as cytolytic cells or by secreting cytolytic and antimicrobial effector molecules such as perforin and granulysin (Stenger et al., 1998; Fletcher and Schrager, 2016). Furthermore, an important requirement for a TB vaccine is to induce significant regional tissue immunity to activate a population of lung-resident immune cells. In fact, an efficient vaccine would need to activate the bactericidal mechanisms within neutrophils to reduce cell-to-cell mycobacterial spread (Fulton et al., 2002). Nowadays, it has been demonstrated that lung neutrophils are directly involved in the activation of T cells in a mouse model of TB infection (Blomgran and Ernst, 2011). In addition, several studies have demonstrated that neutrophils have an important role in the development of different cell types, such as natural killer (Jaeger et al., 2012) and dendritic cells (Schuster et al., 2013). Given that differentiation and expansion of Th17 cells in pulmonary TB is mainly regulated through IL-1β and IL-6-dependent mechanisms (Zhou et al., 2007; Kononova et al., 2015), and the literature demonstrates that neutrophils express and produce cytokines such as IL-1β and IL-6, either constitutively or upon activation by micro-environmental stimuli (Cho et al., 2012; Jaillon et al., 2013), further research will help to improve our understanding of the molecular mechanisms of the vaccine mc^2^-CMX to protect against TB. For example, determine the combinatory signal of IL-1β and IL-6 in neutrophils to act as cofactors for Th17 generation during vaccination against TB. Elucidating the mechanisms of protection associated with Th17 cells, we may be better able to generate rational vaccination strategies toward TB control. Further, detailed mechanism within neutrophils in the induction of different cell types which can be used as multivariate biomarkers such as, natural killer, and CD8+ cytolytic T cells, should be investigated.

## Author contributions

The author confirms being the sole contributor of this work and approved it for publication.

### Conflict of interest statement

The author declares that the research was conducted in the absence of any commercial or financial relationships that could be construed as a potential conflict of interest.
